# Emergency department physicians’ experiences and perceptions with medication-related work tasks and the potential role of clinical pharmacists

**DOI:** 10.1080/17482631.2023.2226941

**Published:** 2023-06-21

**Authors:** Tine Johnsgård, Renate Elenjord, Elin C. Lehnbom, Torsten Risør, Birgitte Zahl-Holmstad, Renata Vesela Holis, Eirik Hugaas Ofstad, Beate Hennie Garcia

**Affiliations:** a Department of Pharmacy, Faculty of Health Sciences, UiT the Arctic University of Norway, Tromsø, Norway; b Hospital Pharmacy of North Norway Trust, Tromsø, Norway; c Department of Health and Caring Sciences, Faculty of Health and Life Sciences, Linnaeus University, Kalmar, Sweden; d Department of Community Medicine, Faculty of Health Sciences, UiT the Arctic University of Norway, Tromsø, Norway; e Department of Public Health, Faculty of Health and Medical Sciences, University of Copenhagen, Copenhagen, Denmark; f Department of Medicine, Nordland Hospital Trust, Bodø, Norway

**Keywords:** Emergency department, physicians, medication safety, experiences, pharmacists, qualitative study, interviews, interprofessional team

## Abstract

**Purpose:**

Medication-related problems are frequent among emergency department patients. Clinical pharmacists play an important role in identifying, solving, and preventing these problems, but are not present in emergency departments worldwide. We aimed to explore how Norwegian physicians experience medication-related work tasks in emergency departments without pharmacists present, and how they perceive future introduction of a clinical pharmacist in the interprofessional team.

**Methods:**

We interviewed 27 physicians in three emergency departments in Norway. Interviews were audio-recorded, transcribed, and analysed using qualitative content analysis.

**Results:**

Our informants’ experience with medication-related work tasks mainly concerned medication reconciliation, and few other tasks were systematically performed to ensure medication safety. The informants were welcoming of clinical pharmacists and expressed a need and wish for assistance with compiling patient’s medication lists. Simultaneously they expressed concerns regarding e.g., responsibility sharing, priorities in the emergency department and logistics. These concerns need to be addressed before implementing the clinical pharmacist in the interprofessional team in the emergency department.

**Conclusions:**

Physicians in Norwegian emergency departments welcome assistance from clinical pharmacists, but the identified professional, structural, and legislative barriers for this collaboration need to be addressed before implementation.

## Introduction

Medication-related problems (MRPs) among emergency department (ED) patients occur frequently and is detrimental for patient care (Budnitz et al., 2011; T. K. Patel & Patel, 2018; P. Patel & Zed, 2002). ED pharmacists contribute significantly to reduce and prevent MRPs (Mekonnen et al., 2016; Mogensen et al., 2012; S. R. Morgan et al., 2018; Roman et al., 2018) and they are highly valued for promoting medication safety and improving patient care (Coralic et al., 2014). Activities performed by ED pharmacists involve e.g., medication reconciliation (MedRec), medication review (MedRev), pharmacotherapy consultation, drug interaction analysis, and patient counselling, as well as other activities like training and educating ED team members (S. R. Morgan et al., 2018).

ED pharmacy services have been established for more than 20 years in the US and UK, which have inspired the development of ED pharmacist practice worldwide (Roman et al., 2018). However, in many countries the ED pharmacist is not a fully integrated part of the health care service. This is the case in Norway, where only a handful EDs have employed pharmacists. During the last decade, MedRec has become an important task in Norwegian hospitals, with both local and national regulations, written procedures, and recommendations (The Norwegian Directorate of Health, 2018; Vorland, 2018), which has increased ED physicians’ workload considerably. At the same time, studies show that 62–84% of medication lists in Norwegian hospitals contain medication discrepancies (Aag et al., 2014; Damlien et al., 2017). This increases the risk of MRPs and challenges patient safety (Makary & Daniel, 2016).

ED pharmacists work closely together with physicians. Literature shows that physicians in primary care settings are generally positive and highly value the contributions of clinical pharmacists in providing comprehensive patient-centred care (Costa et al., 2015; Moreno et al., 2017). A study investigating the collaborative working relationships between pharmacists, physicians, and nurses in an inpatient medical setting found that role clarity and relationships built on mutual respect and trust were essential for successful integration and collaboration with pharmacists (Makowsky et al., 2009). To our knowledge, literature regarding ED physicians’ expectations and perceptions concerning future collaboration with ED pharmacists is scarce. A Swedish study from 2017 investigated perceptions of nurses and physicians before implementing a ward-based clinical pharmacy service (Sjölander et al., 2017). They identified limited experience with and knowledge about what pharmacists can contribute with among these professions, yet positive expectations.

The added value of working in interprofessional teams in healthcare have been established for years (Leape et al., 1999), yet teamwork can be challenging (Zajac et al., 2021). The variability among team members related to e.g., personalities, training, and expert areas, causes differences in understanding and approaching of problems (Hall, 2005). Zajac *et al*. identified numerous internal and external factors influencing team effectiveness, pointing out that “a team of experts does not automatically create an expert team” (Zajac et al., 2021). This is important to keep in mind when planning interventions where new team members, e.g., pharmacists, are introduced.

In the Norwegian “Pharmacist in the Emergency Department” (PharmED) study, the impact of introducing the ED pharmacist as part of the interprofessional team in three EDs in North Norway is being investigated (Vesela et al., 2021). This is a complex intervention, where the overall service provided most likely will change. During the intervention period, ED pharmacists were present as a part of the ED team from 8 am to 7 pm Monday to Friday, performing medication-related tasks according to the need of the patients and the EDs. The primary outcome of the study was “time in hospital during 30 days after admission to the ED”, for which data has not yet been analysed. The project, however, also comprise several sub-studies investigating effects of the ED pharmacists on various outcomes. In this sub-study, we aimed to explore how physicians experienced and perceived medication-related work tasks in the ED *before* the ED pharmacist was introduced, and how they perceived and anticipated the future introduction of the ED pharmacist.

## Methods

### Study design and setting

We conducted semi-structured individual interviews with ED physicians from the three hospitals in Norway where the ED pharmacist was to be introduced in relation to the PharmED study. Annual admission rates in the EDs were in the range of 6 000–16 000 patients, reflecting that the size of the hospitals differs. Physicians in the EDs are employed at different hospital wards, with roster-based shifts in the EDs. Hospital A (urban) is in the university hospital for the Northern part of Norway with more specialized functions than the other two hospitals. Hospital B (urban) is in the smallest hospital, with mainly junior physicians present in the ED, and senior physicians on call in the hospital. Hospital C (urban) is the only hospital with emergency medicine specialists present in the ED to supervise and help junior and senior physicians on call in the ED.

### Interview guide, piloting, and training of interviewers

The research team developed an interview guide informed by the following research questions; 1) Which specific medication-related work tasks are performed by ED physicians? 2) What are ED physicians’ experiences and perceptions with these medication-related work tasks? 3) What are the ED physicians’ perceptions regarding implementing the ED pharmacist? The interview guide (Supplementary file 1) was piloted in one interview and was subsequently modified to make it shorter and more concise while maintaining room for discussion and follow-up questions. Additional questions were also asked during the interviews to get a more elaborate answer and to clarify the interviewers’ understanding. There were three main interviewers, one in each hospital (EF a fifth-year pharmacy student, IN a fifth-year medical student, and TJ a clinical pharmacist and PhD student), see Table I. EF and TJ completed a course in qualitative method at UiT—the Arctic University of Norway and trained on interview skills with healthcare personnel from the ED before conducting interviews with physicians. All three interviewers were trained and supervised by experienced qualitative researchers (ECL, BHG, EHO) during the data collection period.

**Table I.** Overview of interviewers and characteristics of informants.

Alias	Sex^1^	Hospital	Age	Specialty	Seniority	Experience (years)	Main interviewer/ Assistant^2^	Duration of interviews
Walther	M	A	32	Med	Senior	5	EF/TJ	37 min
Ken	M	A	30	Med	Senior	3	EF/TJ	51 min
Adam	M	A	31	Med	Senior	4	EF/TJ	54 min
Christian	M	A	25	Sur	Junior	1	EF/TJ	46 min
Emily	F	A	26	Med	Junior	1	EF/TJ	35 min
Marcus	M	A	28	Sur	Junior	1	EF	41 min
Toby	M	A	36	Med	Senior	3	EF/ECL	62 min
Josephine	F	A	30	Med	Junior	1	EF/TJ	42 min
Nick	M	A	33	Med	Senior	5	EF/TJ	46 min
Irene	F	A	31	Sur	Senior	3	EF/TJ	64 min
Martha	F	B	38	Med	Senior	1	TJ/EF	22 min
Mona	F	B	27	Sur	Junior	1	TJ/EF	39 min
Andrea	F	B	32	Med	Senior	3	TJ/ECL	28 min
Charlotte	F	B	31	Med	Senior	3	TJ/ECL	35 min
Tina	F	B	32	Med	Senior	5	TJ/ECL	44 min
Henry	M	B	30	Med	Junior	1	TJ/BHG	51 min
Vivianne	F	B	27	Med	Senior	3	TJ/BHG	44 min
Christina	F	C	-	-	Junior	>1 year	EHO/IN	32 min
Elias	M	C	-	-	Junior	>1 year	IN	22 min
Martin	M	C	-	-	Junior	>1 year	IN	25 min
Beatrice	F	C	-	-	Junior	>1 year	IN	46 min
Matt	M	C	-	-	Junior	>1 year	IN	44 min
Joey	M	C	-	-	Senior	>1 year	IN	45 min
Celine	F	C	-	-	Senior	>1 year	IN	26 min
Marie	F	C	-	-	Junior	>1 year	IN	22 min
Jacob	M	C	-	-	Senior	>1 year	IN	28 min
Ivan	M	C	-	-	Junior	>1 year	IN	25 min

Note: ^1^*M* = male, F = female.

^2^EF: fifth-year pharmacy student, TJ: pharmacist, IN: fifth-year medical student, ECL: pharmacist, BHG: pharmacist, EHO: physician.

### Data collection

Interviews were conducted from August to November 2019 and took place in meeting rooms at the local hospitals. We aimed duration of 30–45 minutes. Informants in the two largest EDs (hospital A and C) were recruited by a purposive sampling strategy. The interviewers recruited informants in the morning based on the informants’ presence in the ED. We tried to maximize variation in gender, experience, roles, and department specialities classified as medical (med) or surgical (sur) among the informants. In the smallest ED (hospital B), interviews were scheduled beforehand in collaboration with the head of the medical department as the interviewers had to travel to get there. All physicians that were approached accepted participation. We recruited physicians until a sufficient information power was gained in our data (Malterud et al., 2016). No repeated interviews were conducted.

### Data analysis

All interviews were audio recorded and transcribed non-verbatim for analysis by the main interviewers at each hospital. Transcripts were not returned to informants for comments or correction. Audio files were listened to several times to ensure that the transcripts were correct. Each interviewer performed an individual analysis of their empirical data, while the main author (TJ) made the final and overall analysis of all interviews. TJ was supported by experienced qualitative researchers with backgrounds in pharmacy (BHG, ECL) and medicine (TR). Transcripts were read thoroughly several times throughout the analysis, which was inspired by “qualitative content analysis” as described by Graneheim and Lundman (Graneheim & Lundman, 2004). We applied the following five steps during our analysis: 1) Transcripts were read and preliminary categories were noted by the main author who further discussed this with two co-authors, 2) Meaning units were sorted into initial codes using NVivo 12 software 3) Meaning units and codes were transferred to MindManager 2020 software, and meaning units were labelled with more describing codes before further organizing them into subcategories and categories with manifest content. See Supplementary file 2 for an example of coding and categorizing. 4) Categories and subcategories were continuously discussed by the team who agreed upon two main themes with latent content in the final analysis. 5) To verify the analysis, a selection of interviews from each ED was finally read through and coded using the agreed subcategories, categories, and themes. In addition, the individual analyses of the two other interviewers were also reviewed. The entire process was iterative, going back and forth in these steps during the analysis.

### Authors’ preunderstanding

The main researcher (TJ) is a pharmacist, who through both education and experience of working as a clinical pharmacist has gained knowledge about medication use and the potential of MRPs. She believes that pharmacists’ in-depth knowledge about medications and their use should be utilized to a greater extent to increase medication safety and prevent MRPs. The remaining authors have a mixed background from both medicine (TR, EHO) and pharmacy (BHG, BZH, RVH, RE). All are involved in the PharmED study.

### Ethics

The informants were ensured anonymity and complete confidentiality. Transcripts were anonymized, and informants were given a unique code and pseudonyms. Informed consents were obtained from all participants. Quotes used in this article were translated to English by the main author (TJ) and verified by a co-author (ECL). The study was approved by the Data Protection Officer at Hospital Pharmacy of North Norway Trust (nr. 02330).

## Results

### Informants and interviews

We included 27 informants, ten each from the two largest hospitals (A&C), and seven from the smallest (B). The length of the interviews ranged from 22 to 64 minutes. The characteristics of informants are provided in Table I. Unfortunately, age, department speciality and experience were not collected for the physicians from hospital C.

### Themes and categories

**Figure 1.**
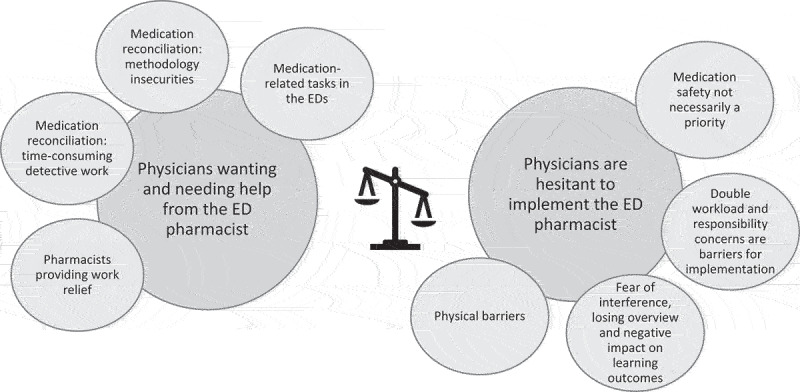
Two themes illustrating how eight categories from the analysis identifies an ambiguity in how the physicians perceive the future ED pharmacist.

During analyses, we identified eight categories which we put together into two themes illustrating the ambiguity identified among the informants; on the one hand they really wanted and needed help, on the other hand they were concerned and hesitant about the pharmacist implementation (Figure 1). As MedRec was a repetitive subject in all interviews despite repetitive attempts to make the informants talk about other medication-related tasks, the categories concern different aspects of MedRec. We did not identify any pattern of differences in view between junior and senior physicians.

#### Medication-related work tasks in the emergency departments

When asked about which medication-related work tasks the informants were performing in the ED, *all* informants explained that their main medication-related work task was to find out which medications a patient uses and to write a medication chart based on this information. They used the term “medication reconciliation” for this task, and most of them expressed something similar to Nick:

I spend a lot of time on medications. […] How I start varies, but I often go into the prescription intermediary (PI; nationwide electronic prescription database) and reconcile the medications there. And then it is not certain that it matches what the patient is using, because they could have paper prescriptions also [not included in the PI], so you have to go and talk to the patient and reconcile the list. […] Patients from nursing home are definitely the biggest challenge […] There is nothing in the PI, and they may come in without a medication list, or a medication list that is outdated. Then you have to search many different systems to create a medication list that is complete and correct, and talk to the patient again, but often you’re left with a feeling that they don’t know either what medications they are using. ***Nick***

When making the informants elaborate on other medication-related tasks performed, it was quite difficult for the physicians to move away from medication reconciliation, but some of them also mentioned “*stopping medications*”, “*starting medications*”, “*checking for drug interactions*”, “*paying attention to risk medications*”. These tasks were done “ad hoc” depending on patients’ characteristics, physicians’ experiences, and time. Other tasks like monitoring for adverse effects, verifying dosages and appropriateness of drugs, or additional medication safety tasks were not mentioned specifically.

#### Medication reconciliation: methodology insecurities

Several different answers were given when we asked what MedRec is and what it means. In the interviews, MedRec was said by some to be about “cleaning up” and getting concordance in the electronic systems, others said it was to find out which medications patients uses, and a few said they did not really know what it was.

MedRec … I feel that I can tick ‘yes’ to MedRec when I have talked to the patient and looked at the PI that it is somewhat correct. Even if it is not correct, you have in a way reconciled [the medication list]. Then you tick ‘yes’ for MedRec and write that it needs to be checked further on the ward if there is some uncertainty about a dose. As long as it is not a complete mess, then I write that I’ve done MedRec. Because it is a part of what you are doing when you check the PI and talk to the patient. ***Christina***

For many informants MedRec was about what they need to do for them to “be allowed” to tick the box for MedRec (as hospital procedures have them do), and not about the patient. Some said it was about having a medication list that is somewhat correct. Beatrice said that she disagreed with those that taught them MedRec from the beginning, and explained:

They say MedRec is when you just go over the medications that they [patients] use and try your best to reconcile what they use regularly and/or as needed. For instance, my colleague says that as long as you have done some sort of assessment [of the medication list], it’s considered MedRec. But to me, MedRec is only performed if the medication list is absolutely correct. You are supposed to get everything right. ***Beatrice***

A challenge expressed by several informants was what they should do if the medication list from the general physician (GP) do not add up with what the patients say. *“One thing is what the lists say, another thing is what the patient says, and a third is what the patient actually does”*, and Jacob explained the dilemma further like this:

It’s a bit problematic when I learn from the patients what they take, because they tell me and they have control over that, but then I see that it doesn’t match the list from the GP that was recently updated. Does that mean that we should start metoprolol, or whatever, even when the patient says they have never used it? Should we trust the medication list or trust the patient? That’s often a problem, I’d say. ***Jacob***

Nobody described MedRec as a standardized systematic *method* for retrieving accurate and complete information about a patient’s current medication use.

#### Medication reconciliation: time-consuming detective work

Informants described and shared frustrations related to the MedRec task, and it was often said to be time-consuming detective work. *“It’s veeeery time-consuming”, “It can take a shitload amount of time”, and “We use a lot of time, and it [MedRec] involves a lot of detective work”* are examples of quotes given (from Mona, Irene, and Vivianne) during the interviews. They expressed that the reason for it being time-consuming is the need for multiple sources of information, and the remaining risk of not being certain about the correctness of the medication list. Charlotte illustrated this by saying:

There is no reliable [medication] list anywhere, there are hundreds of [different] lists. ***Charlotte***

Many informants shared this view and reported that it could be difficult to find out what information that can be trusted. Jacob said that *“the big frustration I see among junior physicians, is that there are so many [medication] lists. We have ours; the GP has theirs; patients have their own; home care nurses have theirs, and it is hard to know which one to trust when things don’t add up. What does the patient take and what should they take?”*. In addition to what Jacob said, informants also mentioned the PI, the Summary Care Record (SCR), post-it notes, phone-calls, next-of-kin, medication lists from nursing homes and pharmacies as potential sources in their detective work.

Informants said that they had different preferences regarding which source to use.

It [PI] is much easier to use than the SCR. But I’m actually not good at using the SCR, I should use it much more. ***Henry***

Other informants expressed that they learned that the gold standard is to use the SCR, and a few admitted to still using mostly the PI. On the contrary, Christina said she thought about the SCR as not being up to date or trustworthy, so she did not use it. Many informants mentioned nursing home patients as being particularly challenging. Henry also said that *“it is a struggle to find out what’s correct”*, and that the medication part is *“often a pain in the ass”*.

#### Positive to work relief provided by pharmacists

Most informants were positive when asked about what their thoughts on adding a pharmacist to the ED interprofessional team was. Mona said she thought it sounded reasonable to add a group of experts on that area early on. It was quite clear among most informants what they need help with, illustrated by the following quote from Vivianne:

What I think we need help with the most is perhaps to get an accurate medication list early on. ***Vivianne***

It was expressed by many informants that this could be a time-saving resource if pharmacists were the ones to do MedRec. Several liked the idea of getting an accurate list served on a silver platter for them to look over. Other tasks informants said they would like help with is *“cleaning up in the electronic health record and PI”* and “*writing the chart*”, in addition they saw the potential to learn from pharmacists and vice versa.

#### Medication safety not necessarily a priority

Many informants expressed that getting an accurate medication list can not be a top priority in the ED. Patients are there in the need of urgent care, and physicians’ focus is to diagnose and treat the patient for the current issue. Several informants said their attitude was to get the list as correct as possible, and expressed something like Vivianne:

In an emergency setting, to be honest, I don’t know if it can be prioritized. To make sure [the medication list] is 100 % correct. ***Vivianne***

It was a general perception among multiple informants that it is ok to postpone completion of the medication list to when the patient has been admitted to the hospital ward, because it is not the same pressure of time there compared to the ED. Multiple informants explained that they felt pressured to clear the ED for patients as soon as possible, both by nurses and because it is measured how much time patients spend in the ED.

The informants also explained that if they do not complete MedRec in the ED, they write in the admission note that MedRec must be done more thoroughly on the hospital ward. At the same time, they also acknowledged that few physicians on the wards prioritize MedRec.

In addition to feeling that MedRec can’t be prioritized in the ED, the informants had the same feeling regarding whether the ED is the best place to have pharmacists. Because of the circumstances physicians work under, like time pressure and heavy workload, it could become a challenge if the ED pharmacist did not quite understand this, and it could also negatively impact patient length of stay in the ED if pharmacists uncovered medication discrepancies in the ED that needed to be clarified before the patient was sent to the ward. One informant said:

If it was one [pharmacist] who was very eager and very thorough, and thought now is the time to make this [the medication list] absolutely perfect, and then spent an extremely large amount of time on it … I don’t think that the pharmacist should be in the ED, but rather on the ward where there is more room to do those deep dives into those things. ***Emily***

When asked if they would want a pharmacist in the ED or on the wards, some informants said that they could see the logic behind the project and placing pharmacists in the ED, but they still believed the ward could be a better place. Christina expressed the following:

If there was a pharmacist who could sort out what [medications] they were coming in with, then maybe. But I think it might be better to do this on the ward. Because there will be medication changes on the ward. Maybe it’s not wise to sort it out when they arrive but rather when they leave … ? I don’t know. ***Christina***

#### Double workload and responsibility concerns are barriers for implementation

Many informants asked during the interviews if pharmacists have access to the PI, and the answer to that is no. They then voiced that without access to the PI the work distribution would not be as straight forward as they initially thought it would be, and this could lead to double workload. This was because physicians would have to check information they received from pharmacists. Several informants raised questions about responsibility and who should do what. Pharmacists do not have the authorization to sign charts and write medication orders or prescriptions, this meant that having pharmacists performing MedRec implied physicians having to sign off on someone else’s work. The following quotes illustrates issues with this work distribution:

I think it’s fair that I sign for medications that I have ordered myself and ensured are correct, but not when I just receive a chart that they [the pharmacists] have checked and printed and I’m just supposed to put my name on it? I do not like that much. Then I’d like to ensure that it’s correct. […] If I were to sign a chart someone had printed and said was correct because they had performed MedRec, then I would have a need for control, to double check. Then I’d prefer them to sign it themselves. ***Andrea***

On my part, if I received a message that ‘MedRec is performed, here is the chart’. Then I’d be suspicious, because I’d feel the need to double check that it had been done [correctly], because it is still me who orders the medications. It is still me who signs the chart. ***Jacob***

Multiple informants raised questions about this during the interviews, and said it was important that role clarification have to be in place before implementing the ED pharmacists. Many informants also expressed medications being physicians’ responsibility, and it is not something they would want the pharmacists to just take over.

#### Fear of interference, losing overview and negative impact on learning outcomes

Some of the informants also voiced a fear that having a pharmacist present could have an impact on their learning outcomes, since some work tasks would be outsourced. They said that they are in the ED to learn, so maybe a different role for pharmacists would be better in that case. Mona and Charlotte said the following:

“[MedRec] is very time-consuming. But with that said, it involves a lot of learning for us. So, I understand in a way when someone thinks it’s dumb that pharmacists would take over this task in the future.” ***Mona***

The junior physicians’ job could be the same, but you [pharmacists] could help me [senior physician] look over [the medication chart]. I think that I over the years have learned a lot by having the role junior physicians have, and that you become more aware of what kind of medications can be “scary” and that you need to keep an extra eye on. ***Charlotte***

They also expressed concerns about losing the overview of the patient if the pharmacist takes over the medication part. Fear that pharmacists would interfere with other medication-related questions were also expressed by multiple informants. Contributing to treatment decisions or giving suggestions to dose adjustments were not roles they thought pharmacists should take on. Tina expressed:

There is something about keeping to your role, and not take part in diagnostics and all that. […] That’s not your job. It might sound a bit harsh, but it’s the way it’s supposed to be. It’s the role of the physician that decides [diagnosis and treatment]. And it has to do with not assuming responsibility for something you shouldn’t even be a part in. ***Tina***

Josephine said she didn’t really know what the pharmacists learns during their education:

I don’t know much about what pharmacists study, but this [the patient] is a human, and there’s the body and that whole package. So, I would think that the physician is probably better suited [to know why the patient uses their medications] than nurses or pharmacists. But I don’t know what pharmacists … What they really do other than being knowledgeable about medications. ***Josephine***

When explained what a pharmacist knows and does, she said that maybe they should use pharmacists more often. A few physicians explained that they knew pharmacists had in-depth knowledge about the use of medications and admitted that they could probably know more about medications than themselves.

#### Physical barriers

The informants also expressed that there were no room or place for the pharmacists in the EDs, as they already had low capacity in their workspace. Multiple informants said there had to be done some reconstruction if there were going to be enough space for pharmacists in the EDs. Two informants said:

No, there’s no space for the pharmacist to sit here [in the ED] and work. There are three computers and many physicians, so we’re already fighting over the computers. ***Charlotte***

I think we need to get a better workspace where there is room for those who work there and for additional staff, because that’s a big challenge right now. So, I think that is an important premise, so you [pharmacists] don’t feel like you come and occupy a workstation and are in the way. ***Adam***

## Discussion

This study provides insight in ED physicians’ experiences with medication-related work tasks, and MedRec was the only medication-related task systematically done for all patients. This indicates that there are room for future clinical pharmacists to systematically contribute with other medication-related tasks in the ED as well, like e.g., MedRev, patient counselling and education of healthcare personnel (Hampton et al., 2022; S. R. Morgan et al., 2018). Our study also provides knowledge about how ED physicians perceive the implementation of a future ED pharmacist. Despite welcoming the ED pharmacist and expressing a positive attitude towards a new collaborating profession, hesitation and concerns were also identified among the informants.

One reason for this contradiction may be founded in the MedRec work task itself, and physicians’ perceptions about it. Our informants fronted many challenges when performing MedRec, for instance lack of time, unreliable information sources and uncertainty about the MedRec methodology, which corresponds with findings in other studies (Al-Hashar et al., 2017; Boockvar et al., 2011; Kleppe et al., 2017). Having dedicated healthcare personnel, like ED pharmacists trained to perform MedRec, could help relieve some of the physicians’ workload (Aag et al., 2014). So, on one hand, informants in our study would value MedRec help from pharmacists.

On the other hand, our informants did not fully see the benefit of MedRec, and perhaps fails to fully understand the pharmacists’ contribution in the ED. This could be because the ED is a high-pace environment where decisions must be made quickly, and our informants stated that MedRec can be postponed to the next day. This aligns with findings by Boockvar et al, who also identified that when time is limited, physicians prioritized other responsibilities over MedRec (Boockvar et al., 2011). Our informants reported that often when they postponed completion of MedRec it was not necessarily done later at the ward either. Similar findings have been reported by Kleppe, where informants found it difficult to gain a complete overview of medications in the ED and thought MedRec was handled on the ward, but were unsure whether this was actually done (Kleppe et al., 2017). Physicians and pharmacists in Boockvar’s study also questioned physicians’ quality of MedRec (Boockvar et al., 2011). Clinical pharmacists are well-trained in medication optimization activities like MedRec and MedRev, and a reconciled medication list is fundamental for an optimal MedRev. Having pharmacists perform these tasks in the ED can identify and prevent MRPs (Rothschild et al., 2010).

Our informants were hesitant to the ED pharmacist contribution, which may be founded in not being fully aware of the clinical pharmacists’ knowledge and competences. This was also found in studies by Sjölander *et al*. and Zielinska-Tomczak *et al*., where participants were unfamiliar with pharmacists and their clinical skills (Sjölander et al., 2017; Zielińska-Tomczak et al., 2021). This contrasts with physicians in the US, having long experience from working with ED pharmacists. A statement issued by the American College of Emergency Physicians (Physicians, 2021), advocates that ED pharmacists serve a critical role ensuring efficient, safe, and effective medication use. However, without this knowledge, it is clearly challenging for physicians to collaborate with and trust pharmacists concerning e.g., treatment decisions or drug choices. In a study of the integration process of clinical pharmacists carried out by Makowsky *et* *al.*, nurses and physicians reported an increased awareness of the clinical role of pharmacists, and said that they learned something more about the knowledge pharmacists have (Makowsky et al., 2009). In order to educate and inform physicians and other healthcare personnel about pharmacists knowledge (and vice versa), interprofessional teamwork should be highly focused on during the undergraduate studies (Green & Johnson, 2015). Having knowledge about each other’s competencies helps build trust, which further could facilitate teamwork, which in the end benefits the patient (Galloway, 2009; Hwang et al., 2017; Makowsky et al., 2009; Radević et al., 2021).

Pharmacists in Norway do not have prescribing rights (The Ministry of Health and Care Services, 1993), and consequently cannot amend medication lists in the hospital system after performing MedRec. Therefore, the ED physicians will have to do the final work and updates on the medication lists themselves and the potential reduction of ED physicians’ work burden will not be fully achieved by the assistance of the ED pharmacist. Additionally, the physician will be holding the final responsibility for any amendments suggested by the ED pharmacist. It is therefore comprehensible that physicians have ambiguous perceptions about the pharmacist contribution. In other countries like UK, Australia, Canada, and Denmark, pharmacists have the legal rights to prescribe or make necessary changes in the medication list if they e.g., uncover medication discrepancies during MedRec (Hoti et al., 2011; Law et al., 2012; Sosabowski & Gard, 2008; Vand et al., 2012).

Our informants feared a potential loss of learning outcome for physicians if pharmacists were to take over tasks from them, like performing MedRec. This is understandable, especially if the ED is not being equipped with pharmacists 24/7. A solution for this may be to employ pharmacist services 24/7, as in other countries (Szczesiul et al., 2009). This debate needs to be fronted within the pharmacy profession in Norway, not being accustomed to work shifts. In order for pharmacists to be fully integrated in positions like the ED, clinical pharmacists must also accept shift work and taking patient responsibility, in accordance with the pharmaceutical care philosophy first fronted by Hepler and Strand (Hepler & Strand, 1990).

The identified ambiguous perception regarding implementation of ED pharmacists indicates a need for a team development program (i.e., simulation training or targeted workshops) to successfully integrate a new team member in the ED interprofessional team during the PharmED study and similar interventions. In a recent study by Morgan *et al*., the impact of an interdisciplinary team development program was evaluated among participants with no previous experience of working together (S. E. Morgan et al., 2021). The program comprised an eight-session workshop and showed meaningful improvements in readiness to collaborate and behavioural trust among participants (S. E. Morgan et al., 2021). Future studies should investigate and evaluate the interprofessional collaboration in the ED, using e.g., the “team effectiveness framework” as described by Zajac (Zajac et al., 2021) or “ten principles of good interdisciplinary team work” as described by Nancarrow (Nancarrow et al., 2013). ED physicians’ experiences of working with ED pharmacists and their perception of appropriate use of resources should also be explored.

### Strengths and limitations

The main strength of this study is the large number of informants with a varied background included in the study. Because of this, we believe that our results may be representative to physicians in other Norwegian EDs, despite involving physicians from only three EDs. It may also be representative to other countries, where the ED pharmacist is not fully integrated. Another strength of this study is that multiple researchers have performed the analyses, which verifies our results. The main limitation to this study is that most interviewers and project participants were also a part of the PharmED project, obviously positive to implementing the ED pharmacist. This may influence the analysis and interpretation of data. However, one interviewer (hospital C) was not a part of the project, and the analysis from those interviews aligned with the overall findings. Results from all three individual analyses aligned with each other, which strengthen our final analysis.

## Conclusion

In this study investigating Norwegian ED physicians’ experiences and perceptions with medication-related tasks and the future introduction of pharmacists in the ED, we found that medication reconciliation was their main focus and concern. They emphasized this task as time-consuming detective work. They warmly welcomed the clinical pharmacist as part of their interprofessional team and expressed a need for assistance. However, they did not seem to know about pharmacist competencies, and were also concerned about professional, structural, and legislative barriers for this collaboration. These barriers must be addressed before future implementation of the ED pharmacist.

## Geolocation information

Geolocation for the three hospitals where the interviews were performed:

Tromsø: Latitude: 69° 38’ 56.04“N, Longitude: 18° 57’ 18.29” E

Harstad: Latitude: 68° 47’ 53.99“N, Longitude: 16° 32’ 29.94” E

Bodø: Latitude: 67° 16’ 48.00“N, Longitude: 14° 24’ 18.04” E

## Supplementary Material

Supplemental Material

## Data Availability

The informants in this study did not give written consent for their data to be shared publicly, so due to the sensitive nature of the research supporting data is not available.
