# Supplementary material for: Emergency department physicians’ experiences and perceptions with medication-related work tasks and the potential role of clinical pharmacists
Source: Int J Qual Stud Health Well-being. 2023 Jun 21;18(1):2226941. doi: 10.1080/17482631.2023.2226941 (PMC10286684; doi:10.1080/17482631.2023.2226941)
Supplement: Supplemental Material [file ZQHW_A_2226941_SM8127.zip › Supplementary files/Supplementary file 1_Semi structured interview guide.docx]

# Supplementary file 1: Semi-structured interview guide

**Describe how you work in the ED regarding medication-related tasks** (introducing question)

*Further questions and keywords for follow-up questions were prompted if necessary:*

- - Obtaining information
  - Discussions with colleagues
  - Considerations when ordering new medications for patients
  - Thoughts around current treatment/adverse drug effects/drug interactions
  - Prescription errors
  - Documentation

What are the pros and cons about the current situation to ensure correct medication use/treatment for patients?

- - What makes you feel safe in the way you work?
  - What could be better?
  - How can medicine safety be improved?

Based on your experiences, what do you think an ED clinical pharmacist should focus upon?

- - Thoughts and experiences with clinical pharmacists/what knowledge they possess?
  - What would physicians like help with?
  - Who should do which tasks? Practical organization

When the clinical pharmacist becomes a part of the ED interprofessional team, how do you think this collaboration should be?

- - What is needed to collaborate well in the ED?
  - Thoughts on bringing in a new profession?
  - Any worries regarding the ED pharmacist?
  - Measures to be taken before implementation?
