# Supplementary material for: Emergency department physicians’ experiences and perceptions with medication-related work tasks and the potential role of clinical pharmacists
Source: Int J Qual Stud Health Well-being. 2023 Jun 21;18(1):2226941. doi: 10.1080/17482631.2023.2226941 (PMC10286684; doi:10.1080/17482631.2023.2226941)
Supplement: Supplemental Material [file ZQHW_A_2226941_SM8127.zip › Supplementary files/Supplementary file 2_data analysis example.docx]

# Supplementary file 2: data analysis example

| Meaning unit | Code | Subcategory | Category |
| --- | --- | --- | --- |
| It [MedRec] takes an awful amount of time.. It is really… It can take 45 minutes just to make up the chart. | MedRec takes a long time to perform, chart-making as well | Challenging and time-consuming work | MedRec often feels like time-consuming detective work |
| We use a lot of time | A lot of time is spent |  |  |
| If it is paracetamol and calcium it is something else, but the multimorbid medical patients use a lot of medications, so it takes a lot of time | A couple of drugs per patient is ok, but multimorbidity increases time spent on MedRec for a patient |  |  |
| Sometimes when you have little time, you get like drained and tired and think that I don’t have time for this now | Time is limited, and MedRec takes time that you don’t have, which is frustrating |  |  |
| It is problematic that it [MedRec] sometimes takes a lot of time | Problematic that MedRec takes time |  |  |
| It takes an awful amount of time to clean up in medication lists, obtain sources and retrieve information | The MedRec process takes time |  |  |
| There is no reliable list anywhere, there are hundreds of lists | Many available sources for information make it hard to know what is correct | Detective work |  |
| So, it is like detective work | MedRec involves detective work |  |  |
| We are interested in information about it, but it is a rocky road | Medication information is interesting, but the path is difficult |  |  |
| We use a lot of time, and it [MedRec] involves a lot of detective work | MedRec takes time because it is like detective work |  |  |
| The big frustration that I see in junior physicians, is that there are so many [medication] lists. We have ours, the general physician has his, patients have their own, home care nurses have theirs, and it is hard to know which one to trust when things do not add up. What does the patient take and what should he take? | Many sources of information make it hard to know what to trust and find out what is correct |  |  |
